# Safety and effectiveness of propranolol in severely burned patients: systematic review and meta-analysis

**DOI:** 10.1186/s13017-017-0124-7

**Published:** 2017-03-02

**Authors:** Ramiro Manzano-Nunez, Herney Andrés García-Perdomo, Paula Ferrada, Carlos Alberto Ordoñez Delgado, Diego Andrés Gomez, Jorge Esteban Foianini

**Affiliations:** 1 Clinical Research Center, Fundación Valle del Lili, Cali, Colombia; 2 UROGIV Research Group, Universidad Del Valle, Cali, Colombia; 3 Surgical Critical Care, Virginia Commonwealth University, Richmond, VA USA; 4 Division of Acute Care Surgery, Fundación Valle del Lili, Cali, Colombia; 5 Division of Surgery, Clinica Foianini, Av. Irala 468, Santa Cruz de la Sierra, Bolivia

**Keywords:** Propranolol, Burns, Pharmacology, Critical care, Adrenergic antagonist

## Abstract

**Background:**

The objective of this systematic review was to determine the effectiveness and safety of propranolol compared to placebo or usual care for improving clinical relevant outcomes in severely burned patients (TBSA >20%).

**Methods:**

Relevant articles from randomized controlled trials were identified by a literature search in MEDLINE, EMBASE, and CENTRAL. We included trials involving patients with a severe burn (>20% of total body surface area affected). Trials were eligible if they evaluated propranolol and compared to usual care or placebo. Two investigators independently assessed articles for inclusion and exclusion criteria and selected studies for the final analysis. We conducted a meta-analysis using a random-effects model.

**Results:**

We included ten studies in our systematic review. These studies randomized a total of 1236 participants. There were no significant differences between propranolol and placebo with respect to mortality (RD −0.02 [95% CI −0.06 to 0.02]), sepsis (RD −0.03 [95% CI −0.09 to 0.04]), and the overall hospital stay (MD −0.37 [−4.52 to 3.78]). Propranolol-treated adults had a decrease in requirements of blood transfusions (MD −185.64 [95% CI −331.06 to −40.43]) and a decreased heart rate (MD −26.85 [95% CI −39.95 to −13.75]).

**Conclusions:**

Our analysis indicates that there were no differences in mortality or sepsis in severely burned patients treated with propranolol compared with those who had usual care or placebo. However, the use of propranolol in these patients resulted in lower requirements of blood transfusion and lower values of heart rate. The evidence synthesized in this systematic review is limited to conclude that propranolol reduces the length of hospital stay among severely burned patients. Future trials should assess the impact of propranolol on clinically relevant outcomes such as mortality and adverse events.

**Electronic supplementary material:**

The online version of this article (doi:10.1186/s13017-017-0124-7) contains supplementary material, which is available to authorized users.

## Background

Burn injuries are among the most severe of all injuries [1] with approximately 90% of cases occurring in low- and middle-income countries [2]. Burns are a common cause of morbidity and mortality, and most occur in a domestic setting with children from birth through 4 years having the highest burden of the condition. Episodes occur commonly at home and principally due to scalds, hot objects, and fires [3, 4]. For adults, the incidence of burns is low until the 30s, with cases occurring at home, outdoors, and at workplaces in equal proportions [1]. The incidence of burns in low- and middle-income countries is 1.3 per 100,000 population whereas in high-income countries is approximately of 0.14 per 100,000 population with burn injuries ranking the top 15 leading causes of burden of disease globally [2, 5].

Burn injuries covering more than 20% of the total body surface area (TBSA) cause an inflammatory and subsequent hypermetabolic response that starts immediately post-burn and can persist for years [6, 7]. Hypermetabolic state has two aspects—the “ebb” and “flow” phases. The ebb phase, occurring within the first 48 h after injury, is associated with decreased cardiac output, oxygen consumption, and metabolic rate. The chronic “flow” phase is a critical phase that requires medical intervention to reduce the risk of fatal outcomes [8].

The release of cytokines and other pro-inflammatory mediators at the site of injury has a systemic effect once the burn reaches 20% of total body surface area. Injuries greater than this percentage invariably results in severe impairments of cardiovascular, respiratory, metabolic, and immunological functions derived from hypermetabolic changes [9]. The hypermetabolic response along with catecholamines and corticosteroids increase liver and cardiac work, impair muscle function, increase the risk of sepsis, and produce hormonal abnormalities that augment morbidity and mortality [6, 9–11].

Several interventions have been proposed to decrease the hypermetabolic response and to improve outcomes in the burned patient [12–16]. Propranolol through β-adrenergic receptor blockade has been proposed as an effective strategy for reducing post-burn catabolism and therefore to improve outcomes in burned patients. The objective of this systematic review was to determine the effectiveness and safety of propranolol compared to placebo or usual care for improving clinical relevant outcomes in severely burned patients (TBSA >20%).

## Methods

This systematic review and meta-analysis was conducted according to the recommendations of the Cochrane Collaboration and following the PRISMA Statement. The PROSPERO registration number is CRD42016042230.

## Inclusion criteria

We included only randomized controlled trials (RCTs) involving patients with a severe burn, defined as a burn with an affected area greater than 20% of the total body surface area (TBSA). Trials were eligible if they evaluated propranolol as the intervention of interest in adults or children and compared to usual care or placebo. We excluded trials where propranolol was compared to other pharmacologic interventions and trials evaluating the combination of propranolol with other interventions. Two investigators independently assessed articles for inclusion and exclusion criteria and selected studies for the final analysis, with divergences finally resolved by consensus.

## Outcomes

We prespecified clinical relevant outcomes as our primary outcomes. Those included mortality, adverse events, need for transfusions/blood loss, the occurrence of infections/sepsis, and length of hospital stay. Secondary outcomes were heart rate, hypertriglyceridemia, and hyperglycemia. Only those trials providing sufficient information (i.e., measures of treatment effects and the associated precision) were included in our statistical analysis.

## Search methods

Literature search strategy was built according to current recommendations (Additional file 1: Table S1) [17–19]. We searched MEDLINE, EMBASE, and CENTRAL from inception to 2016. We also hand-searched references from relevant narrative reviews and previous systematic reviews for more trials. Other sources were thesis databases, OpenGrey and Google Scholar. Authors were contacted to complement data by e-mail. No language restrictions were used.

## Study selection and data collection

Two individuals independently assessed the titles and abstracts identified by the searches for potential eligibility, and the full-text articles were retrieved for those that appeared relevant. Two investigators independently assessed full-text articles for final eligibility. Disagreements were resolved by consensus or by a third, independent reviewer. The following information was independently extracted using a standardized form: study design, geographic location, authors names, title, objectives, inclusion and exclusion criteria, the number of patients included, losses to follow-up, the definition of interventions, definitions of outcomes, outcomes measures, funding, and status of data on clinical trials website.

## Risk of bias

The internal validity of each trial included in this review was critically evaluated for bias according to the Cochrane Collaboration tool for assessing the risk of bias [20] which covers sequence generation, allocation concealment, blinding, incomplete data, selective reporting, and other biases. Two independent investigators made a judgment about the possible risk of bias from extracted information, rated as “high risk,” “low risk,” or “unclear risk”. We computed a graphic representation of potential bias using RevMan 5.3.

## Data analysis/synthesis of results

A meta-analysis was performed to assess the overall outcomes of propranolol compared to usual care or placebo. The statistical analysis was performed using Review Manager 5.3 (RevMan® 5.3). For dichotomous outcomes, we extracted data on the total number of participants, the number that experienced the outcomes, and the number analyzed. For continuous outcomes, we extracted end-value means with standard deviations (SD). Whenever possible, we used results from an intention-to-treat population.

Mean differences (MD) and risk differences were pooled using a random-effects model. The results were reported in forest plots of the estimated effects of the included studies with a 95% confidence interval (95% CI). Meta-analysis was considered since the included studies were similar in terms of participants, interventions, and outcomes (clinical homogeneity). Heterogeneity was evaluated using the *I* ^2^ test. For the interpretation, it was determined that the values of 25, 50, and 75% in the *I* ^2^ test corresponded to low, medium, and high levels of heterogeneity, respectively.

## Results

**Fig. 1:**
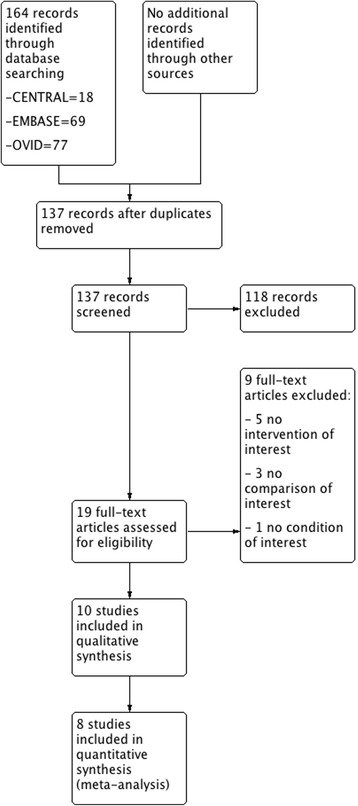
Flowchart according to PRISMA guidelines

We identified 164 records from our searches, of which 19 trials were eligible to be included in our systematic review. After applying inclusion and exclusion criteria, ten studies [21–30] were included in the systematic review, all of them in the qualitative synthesis and eight in quantitative synthesis (meta-analysis). Figure 1 shows the flowchart for the selection of randomized trials.

## Characteristics of included studies

Included trials were published between 2001 and 2015. These trials randomized a total of 1236 participants with a sample size ranging from 25 to 406 (median, 76; interquartile range, 58.5–195.5). Five trials (50%) [22–25, 27] were single-center trials. Of the ten RCTs included, nine recruited participants from the USA and one trial recruited patients from Iran [27]. Losses to follow-up were reported in four trials [22–24, 29], and finally, an intention-to-treat analysis was performed in two trials [22, 24]. Main characteristics from included studies were summarized in Additional file 2: Appendix 1.

Six RCTs were done in adults and four were done in children [23–25, 29]. Regarding how the condition was defined, four RCTs [23, 25, 26, 28] included patients with burns greater than 40% of TBSA, five [21, 22, 24, 29, 30] included patients with burns greater than 30% of TBSA, and one trial [27] included patients with burns between 20 and 50% of TBSA. Interventions and comparators were very similar, with propranolol (at similar doses) and placebo or usual care being the intervention and comparator, respectively, in all trials. The similarity in interventions and comparators between trials could result in low clinical diversity at this point. Main characteristics of participants from individual RCTs are summarized in Table 1.

**Table 1:** Characteristics of participants. Age is described as mean and standard deviation

No.	Study	Group	Total number of randomized patients	Age	TBSA (%) of patients to be included	Country	Socio-demographics
1	Williams 2011 [29]	Propranolol	125	7 (5)	Burns >30%	USA	Not described
		Standard care	215	8 (5)			
2	W Norbury 2007 [28]	Propranolol	33	Not described	Burns >40%	USA	Not described
		Standard care	33	Not described			
3	Wurzer 2015 [30]	Propranolol	43	Not described	Burns >30%	USA	Not described
		Standard care	39				
4	Ali 2014 [21]	Propranolol	21	Not described	Burns >30%	USA	Not described
		Standard care	21				
5	Komak 2012 [26]	Propranolol	29	Not described	Burns >40%	USA	Not described
		Standard care	35				
6	Ali 2015 [22]	Propranolol	35	41 (14)	Burns >30%	USA	Not described
		Standard care	34	38 (16)			
7	Herndon 2012 [24]	Propranolol	90	7 (5)	Burns >30%	USA	Majority of participants were Hispanic: 97% in the control group and 91% in the propranolol group
		Standard care	89	7 (5)			
8	Akbar Mohammadi 2009 [27]	Propranolol	37	27.21 (9.73)	Burns 20–50%	Iran	Not described
		Standard care	42	24.54 (12.06)			
9	Jeschke 2007 [25]	Propranolol	102	7.2 (0.6)	Burns >40%	USA	Not described
		Standard care	143	7.8 (0.4)			
10	Herndon 2001 [23]	Propranolol	12	6.6 (1.5)	Burns >40%	USA	Not described
		Standard care	12	7.8 (1.4)			

With respect to outcomes (see Additional file 1: Table S1), four trials [22, 24, 25, 27] presented mortality data, in three trials [21, 22, 27], the quantity of blood transfused was reported, three trials [23, 25, 27] measured occurrence of sepsis, five trials [22, 24, 25, 27, 29] reported length of hospital, eight trials [21–24, 26, 28–30] defined hemodynamic parameters such as heart rate, mean arterial pressure, cardiac output, and/or stroke volume as part of the outcomes measured. Three trials [21, 22, 27] presented data about wound healing in terms of the number of grafting procedures and the average time between grafting procedures, and only one trial [22] presented data about adverse effects of propranolol.

## Risk of bias

**Fig. 2:**
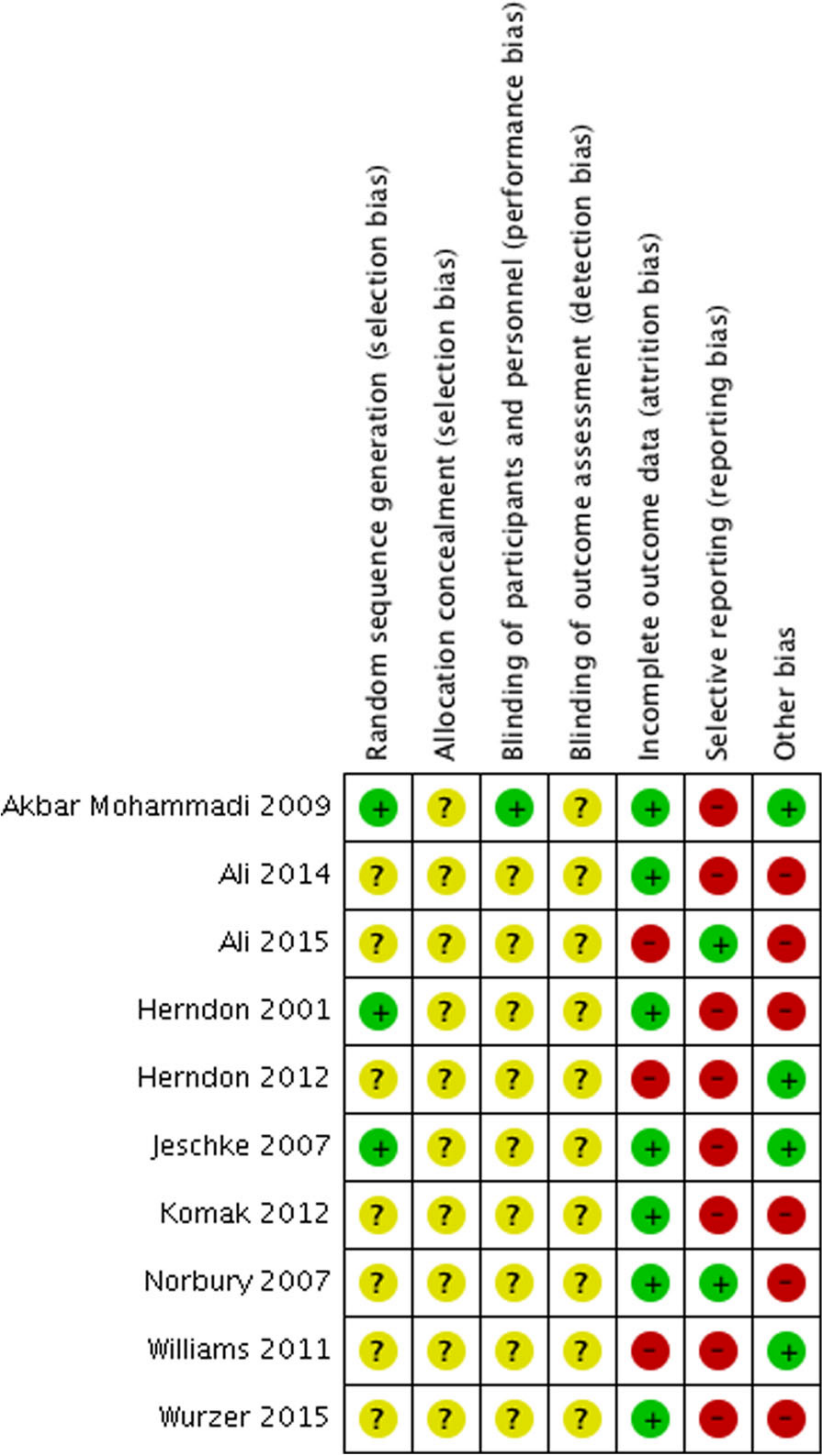
Risk of bias from individual study

**Fig. 3:**
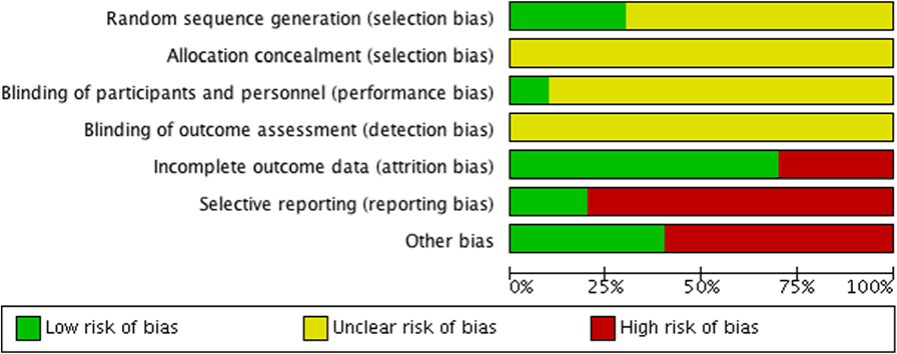
Risk of bias across studies

Seven (70%) and ten trials (100%) were rated as unclear risk of bias for random sequence generation and allocation concealment, respectively; the remaining three trials (30%) from the random sequence generation domain were rated at low risk of bias. Only one trial (10%) reported masking of study participants, and the remaining nine trials (90%) were rated at unclear risk of bias for this domain. All trials were rated at unclear risk of bias in terms of blinding of outcome assessors. Three trials (30%) were rated as high risk of attrition bias because of losses to follow-up. High risk of selective reporting was found in eight trials (80%); thus, a dangerous quantity of reporting bias could have been introduced from those trials but it was difficult to estimate the likely magnitude and the likely direction of this bias. High risk of other biases was found in six trials (60%) due to small sample sizes. Five trials (50%) reported receiving funding for the research, and none was funded by pharmaceutical industry. Finally, none of the trials posted results in clinical trials website (https://clinicaltrials.gov/) and six trials reported receiving ethical approval. The overall risk of bias of the included RCTs is best represented in Figs. 2 and 3.

## Outcomes

**Fig. 4:**
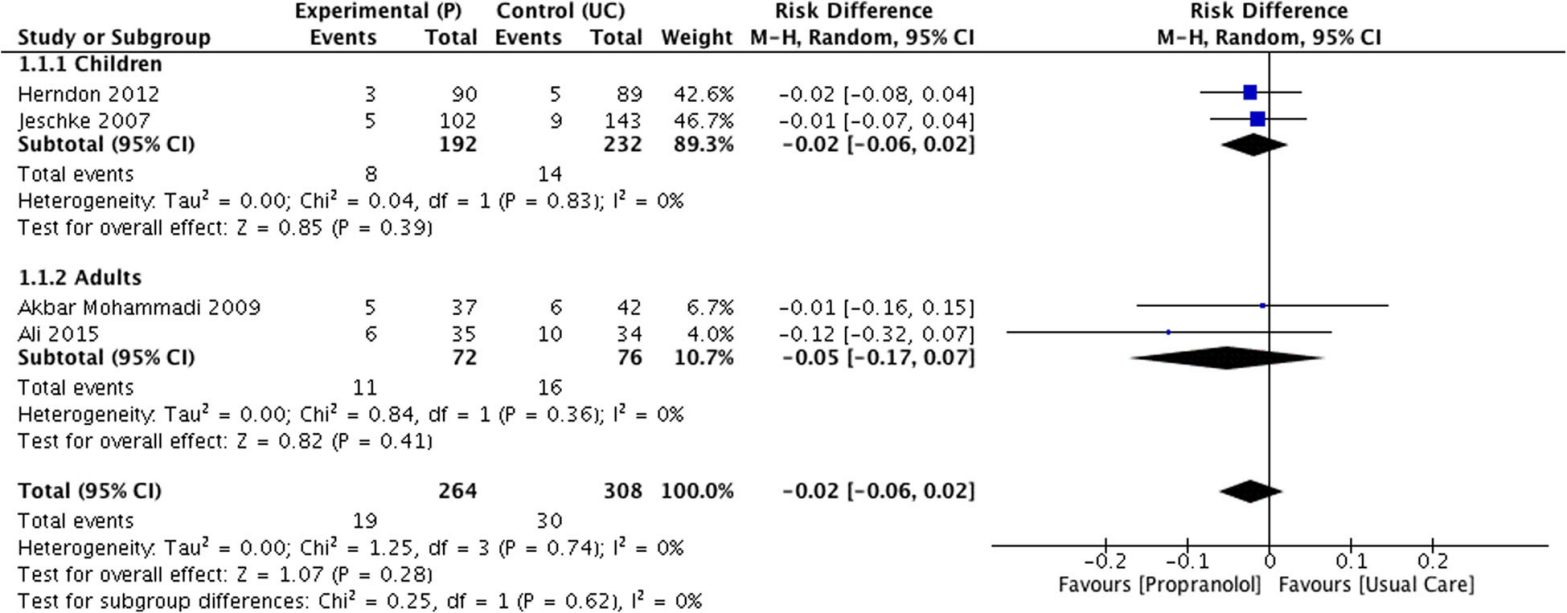
Forrest plot for mortality (propranolol vs usual care)

**Fig. 5:**
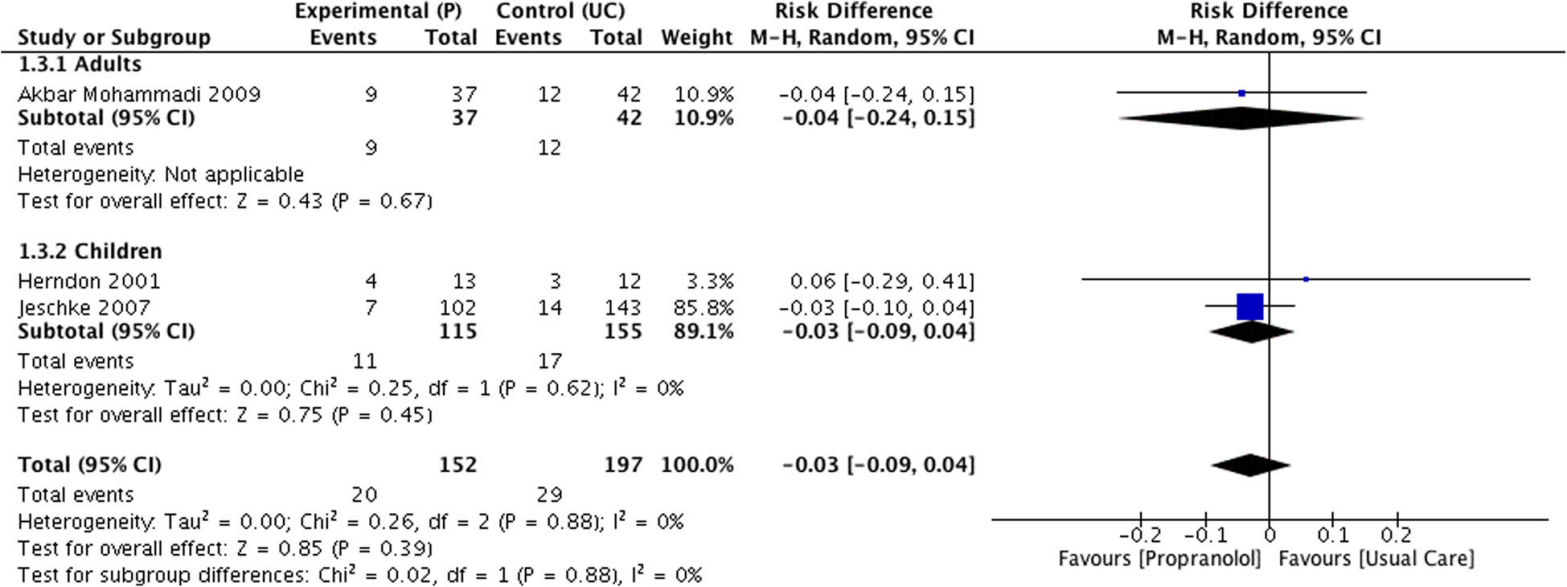
Forrest plot for sepsis (propranolol vs usual care)

There were no significant differences between propranolol and placebo/usual care with respect to mortality (RD −0.02 [95% CI −0.06 to 0.02]), and also, no differences were found in the sub-group analysis when assessing this outcome in children (RD −0.02 [95% CI −0.06 to 0.02]) and adults (RD −0.05 [95% CI −0.17 to 0.07]) (Fig. 4). Similarly, no significant differences were found when assessing overall occurrence of sepsis (RD −0.03 [95% CI −0.09 to 0.04]) with no differences in children (RD −0.03 [95% CI −0.09 to 0.04]) and adults (RD −0.04 [95% CI −0.24 to 0.15]) (Fig. 5). When assessing the length of hospital stay, propranolol did not shorten the overall number of days of hospitalization when compared to usual care or placebo (MD −0.37 [−4.52 to 3.78]) but this was not sustained when assessing sub-groups. In the sub-group analysis, the use of propranolol was associated with a lower length of hospital stay in adults (MD −6.59 [95% CI −10.18 to −3.0]), a fact that did not occur in children (MD 2.30 [95% CI −3.36 to 7.96]).

With respect to blood transfused, propranolol was associated with a lower amount of transfused blood (measured in milliliters) in adults (MD −185.64 [95% CI −331.06 to −40.43]). Propranolol was associated with lower values of heart rate (beats per minute) when compared to usual care (MD −26.85 [95% CI −39.95 to −13.75]) and this was consistent in adults (MD −32.54 [95% CI −38.45 to −26.63]) and children (MD −19.0 [95% CI −31.12 to −6.88]).

Finally, it is important to mention that studies done in children did not report data about transfused blood. On the other hand, some of our primary outcomes are not reported and this is because primary research did not report data on hypertriglyceridemia and hyperglycemia.

## Sensitivity analysis

Of the ten studies included in our systematic review, eight were rated as high risk for selective reporting; therefore, we decided to exclude those studies from our quantitative synthesis to evaluate the effect of this bias on results. We found that there were no differences in mortality (RD −0.12 [95% CI −0.32 to 0.07]) and quantity of blood transfused (MD −405 [95% CI −1343.48 to 533.48]) when comparing propranolol and usual care after excluding studies with high risk of selective reporting.

On the other hand, three of the ten studies were rated as high risk for attrition bias; thus, after excluding those studies from quantitative results, we found that propranolol was associated with a lower length of hospital stay in children (MD −2.0 [CI 95% −2.51 to −1.49]) and adults (MD −6.54 [95% CI −10.19 to −2.89]).

## Discussion

The present study was designed to determine the effectiveness and safety of propranolol, compared to placebo or usual care, for improving clinical relevant outcomes in severely burned patients. In summary, no significant differences were found between propranolol and usual care in terms of mortality and sepsis occurrence. Propranolol-treated adults had a decrease in requirements of blood transfusion and a decreased heart rate. There was no difference in the overall length of hospital stay. However, the subgroup analysis showed decreased hospital stay in adults treated with propranolol. Furthermore, the sensitivity analysis revealed significantly decreased hospital stay in both adults and children treated with propranolol.

Severe burn injuries cause a hypermetabolic response driven by supraphysiologic elevations in stress hormones, catecholamines, and inflammatory mediators [6, 9, 11]. The hypermetabolic response is characterized by increased peripheral lipolysis, increased muscle wasting, elevated resting energy expenditure, and suppressed immune function. This response impedes recovery or leads to organ failure and death [9]. Therefore, the hypermetabolic response can be seen as an intermediate variable in the causal pathway between burns and relevant outcomes in burned patients; and thus, it is not irrational to think that the use of propranolol, by mitigating the deleterious effects of the hypermetabolic response, can result in improved outcomes among severely burned patients.

Burns impose a significant challenge and are associated with mortality rates between 1.4 and 18% with age groups and greater percentage total body surface area (TBSA) affected being associated with greater fatality rates [31–33]. It is estimated that 265,000 deaths every year are caused by burns, with the higher proportion of these fatalities occurring in low- and middle-income countries [2, 34], places where burns are a leading cause of mortality and a public health problem; thus, interventions to reduce mortality in burned patients could be of great value. Our study found that the use of propranolol did not significantly reduce mortality in patients with severe burns, a result that is consistent with previous research [24].

Burns are associated with changes in the immune response. Severe burns induce a state of immunosuppression [35] that predisposes burn patients to infectious complications and sepsis [36]. In theory, the use of β-adrenergic blocking agents could improve immune status through the suppression of catecholamine-mediated hypermetabolism in critically ill patients. However, the potential immunological side effects of the β-adrenergic blockade are not well elucidated. Furthermore, research in animal models has found that the use of pharmacologic β-adrenergic blockade during sepsis is associated with disturbances in the immune system that predisposes to organ dysfunction and associated mortality [37, 38]. The current study found no significant differences between propranolol and placebo/usual care when measuring sepsis occurrence.

Overall, the use of propranolol in severely burned patients was not associated with a lower length of hospital stay. However, after performing subgroup and sensitivity analyses, the use of propranolol was associated with a lower length of hospital stay (LOS) in severely burned adults and children. This finding could be of great importance if we take into account that burn patient management requires significant financial resources, and LOS has a large impact on cost. It is not clear how propranolol could reduce the length of stay since many factors are related to this outcome. Such factors include the area and deepness of the burn, the sex and age of the patient, and the time until the initiation of the treatment [39, 40]. Although reductions of time spent in the hospital will reduce costs, and therefore are of great importance for managerial attention [41], our results provide limited evidence to conclude that propranolol decreases the length of hospital stay.

The sustained elevation of catecholamines after severe burn injuries may be detrimental to the myocardium. Severe burns may cause cardiac deficiency, myocardial hypoxia, and cardiac death [42]. In this setting, propranolol has been proposed as an intervention aimed to improve the hemodynamic state of severely burned patients. Our pooled analysis showed, as expected, that propranolol reduced heart rate in severely burned patients. This result seems to be consistent with other research which found that the administration of propranolol improves cardiac physiology and reduces cardiac stress among patients with severe burns [43, 44]. For example, Brown et al. [44] reported a 22% decrease in resting heart rate after 2 weeks of propranolol treatment in burned adults. Herndon et al. showed a 20% decrease in baseline heart rate with the use of propranolol in burned children [23]. Although the use of propranolol in severely burned patients can improve cardiac physiology, it is also associated with frequent episodes of hypotension and bradycardia [44].

Another important finding was that propranolol-treated adults had a decrease in requirements of blood transfusion. A possible explanation for this result might be that the use of propranolol in severe burns improves wound healing and therefore decreases the number of skin graft procedures [27]. In major burns, the blood loss from one debridement and grafting can be enormous. Such losses can result in cardiovascular derangements and even shock. Therefore, decreasing the number of skin graft procedures reduce the chance for major bleeding. Even if propranolol can decrease requirements of blood transfusion, it is important to keep in mind that several factors influence the decision to transfuse a burn patient and also several factors are associated with transfusions thresholds. A previous study explored the relationship between patient characteristics and number of transfusions. This study found that the number of PRBC and plasma transfusions was significantly influenced by TBSA burn, the presence of co-existing inhalation injury, and the type of anticoagulation used [45]. Palmieri and Greenhalgh [46] used a survey to identify burn center physician blood transfusions practices. They found that inhalation injury influenced the decision to transfuse blood and that the most frequent reasons for transfusions were hemorrhage, anemia, hypoxia, and cardiac disease.

A key component of evidence-based medicine is the knowledge about the balance of benefit to harm. Therefore, we must acknowledge that only one [22] of the trials reported adverse events of propranolol and that was one of the reasons why we rated eight trials as having a high risk for selective reporting. Adverse events reported in the trial by Ali et al. [22] included bradycardia, bradypnea, hypotension, and ischemia. Brown et al. [44] studied the safety of propranolol use in adult patients with burn injuries. In this study, the use of propranolol was associated with frequent episodes of hypotension and bradycardia. The authors concluded that despite the potential beneficial effects, burn care providers must recognize the potential iatrogenic hemodynamic effects of propranolol. Previous research on other conditions in children and adults have found that propranolol is associated with potentially life-threatening reactions such as pulmonary edema, shock, or complete heart block [47–49].

The most important limitation of this systematic review lies in the fact that only one study utilized mortality as a primary outcome [27]. From an orthodox view, evaluation of mortality in this study is preliminary as the remaining studies reporting mortality data are underpowered for this outcome. However, systematic reviews should include all outcomes that are likely to be meaningful to stakeholders even if those are not reported as primary endpoints in the primary research [20].

To develop a full picture of the effectiveness of propranolol on burns, additional studies will be needed that include clinical relevant outcomes, such as mortality as their primary outcomes. On the other hand, as only one trial reported data on adverse effects of propranolol, a further study with more focus on the safety of propranolol is therefore suggested.

## Conclusions

Propranolol has been used as a therapy to reduce the deleterious effects of hypermetabolic response. Our analysis indicates that although lower in propranolol-treated patients, mortality and sepsis were not significantly different between compared groups. However, the use of propranolol in severely burned patients resulted in lower requirements of blood transfusion and lower values of heart rate. On the other hand, the evidence synthesized in this systematic review is limited to conclude that propranolol reduces the length of hospital stay among severely burned patients. Future trials should assess the impact of propranolol on clinically relevant outcomes such as mortality and adverse events.

## Additional files

Additional file 1: General characteristics of included studies. (DOCX 18 kb)
Additional file 2: Appendix 1. Search strategy. (DOCX 51 kb)

## Abbreviations

TBSA: Total body surface area
