# Supplementary material for: Safety and effectiveness of propranolol in severely burned patients: systematic review and meta-analysis
Source: World J Emerg Surg. 2017 Mar 2;12:11. doi: 10.1186/s13017-017-0124-7 (PMC5335497; doi:10.1186/s13017-017-0124-7)
Supplement: Additional file 1: — General characteristics of included studies. (DOCX 18 kb) [file 13017_2017_124_MOESM1_ESM.docx]

| **Study / Total Study Duration** | **Objective** | **Inclusion criteria** | **Type of study (MC-SC) / Method of randomization** | **Interventions** | **Comparators** | **Outcomes** | **Intention to treat / Ethical Approval / Funding / Data published in clinicaltrials.gov** | **Conclusion of study authors** |
| --- | --- | --- | --- | --- | --- | --- | --- | --- |
| Williams 2011 / 1998-2007 | The aim of this study was to determine in a large, prospective, randomized, controlled trial the dose of propranolol per kilogram of body weight that would elucidate the appropriate attenuation of CW during the acute hospitalization and the associated dose kinetics of the drug. | Burns over 30% TBSA who consented to an institutional review board-approved experimental protocol between 1998-2007 and were admitted to our burn unit and required ≥1 operative intervention | Randomized controlled trial (Not described) / Not described | Propranolol dose was initiated at 1mg/kg per day (enterally) and titrated to decrease heart rate by 15-20% of admission heart rate. It was given once patients were fluid stabilized. | Standard Care, not described by authors | Heart rate; mean arterial pressure; cardiac output; cardiac index; stroke volume | No/ Yes/ Yes, described. No funding by pharmaceutical industry/ No | Beneficial decrease in heart rates to 10% when propranolol is given at 1mg/kg/d |
| W Norbury 2007 / Not described | Determine the effect of propranolol on infections and clinical parameters during the acute phase post-burn. | Not described | Randomized controlled trial (Not described) / Not described | Standard care plus propranolol (0.5-1.5mg/kg every 6h) | Standard Care, not described by authors | Heart rate; resting energy expenditure; incidence of infection; IGF-I; IGFBP-3; GH, prealbumin; CRP | No/ Not described/ Not described/ No | Propranolol attenuates infections, inflammatory markers and fatty acids levels while improving cardiac work and endogenous anabolic hormone levels. We suggest that propranolol is safe and efficacious modulator of the post-burn response |
| Wurzer 2015 2005-2012 | Quantify the impact of propranolol on cardiac stress measures by the PiCCO system in severely burned children | Children with burns over 30% TBSA and up to 18 years of age | Randomized controlled trial (Not described) /Not described | Propranolol 4mg/kg/d | Standard Care, not described by authors | Cardiac index, cardiac output, extravascular lung water index, heart rate, mean arterial pressure, systemic vascular resistance index, stroke volume and cardiac work | No/ Not described/ Not described/ No | In this study we show, using PiCCO, that propranolol reduces the hyperdynamic circulatory response and therefore cardiogenic stress, according to severe burn injury in children. Although propranolol did not adversely affect peripheral perfusion, wound healing and complication rates. |
| Ali 2014/ Not described | The purpose of this study was to investigate perioperative effects of propranolol in severely burned adults | Patients with >30% TBSA | Randomized controlled trial (Not described) / Not described | Propranolol 0.54mg/kg | Standard Care, not described by authors | Heart rate, number of days between grafting procedures | No/ Not described/ Not described/ No | The current data demonstrated diminished blood loss during surgery in severely burned patients on propranolol. Whether propranolol safely attenuates the hyperdynamic and hypermetabolic response as measured by clinical outcomes remains yet to be determined in adults |
| Komak 2012 / 2005-2008 | We examined the effect of propranolol on cardiovascular parameters derived from transpulmonary thermodilution measurements in severely burned children | Pediatric patients with >40% TBSA burns | Randomized controlled trial (Not described) /Not described | Propranolol (no description of dosis) | Standard Care, not described by authors | Mean arterial pressure, heart rate, cardiac index, intrathoracic blood volume index and extravascular lung water | No/ Not described/ Not described/ No | Blocking the hyperdinamic response with beta-antagonist decreases cardiac work and overall energy expenditure. |
| Ali 2015 / 2004-2014 | The purpose of this study was to investigate whether wound healing and perioperative hemodynamics are affected by propranolol administration in severely burned adults | Older than 18 years, burn wounds covering ≥30% of TBSA, treatment with at least one surgical skin grafting procedure, and consent to participate in data collection | Randomized controlled trial (SC) /Not described | Propranolol 3mg/kg/d | Standard Care, not described by authors | Heart rate, wound healing (average number of skin grafting procedures, time between skin grafting procedures), blood loss, fluid balance, adverse events | Yes/ Yes/ Yes, described. No funding by pharmaceutical industry/ No | In severely burned adults, administration of propranolol during acute hospitalization diminishes blood loss during skin grafting procedures and speeds wound healing |
| Herndon 2012 / 2002-2011 | To determine the safety and efficacy of propranolol given for 1 year on cardiac function, resting energy expenditure, and body composition in a prospective, randomized, single-center, controlled study in pediatric patients with large burns | Study patients, 0.3 to 18 years of age ar the time of injury, had more than 30% total body surface area burns and required 1 or more surgical interventions. | Randomized controlled trial (SC) /Not described | Propranolol 4mg/kg/d | Standard Care, described by authors | Heart rate, blood pressure, energy expenditure (indirect calorimetry); body composition; hormone levels (PTH, IGF-I;osteocalcin; testosterone); level of proteins and albumin | Yes/ Yes/ Yes, described. No funding by pharmaceutical industry/ No | Propranolol is safe and markedly decreases heart rate and cardiac work. This treatment decreases central body mass, reduces truncal fat, and improves lean body mass and bone mineral density. |
| Akbar Mohammadi 2009 / 12 months | To investigate the clinical effects of propranolol on burn wound healing | Patients with burn size 20 to 50% of the TBSA and between 16 and 60 years of age | Randomized controlled trial (SC) / Random number-generating scheme | Propranolol 1mg/kg/d - increased gradually to maximum dose of 1.98mg/kg/d | Standard Care, not described by authors | Mortality, sepsis; Wound healing | No/ Yes/ Not described/ No | Administration of propranolol, improved burn wound healing, and decreased healing time and hospital stay period. The use of propranolol decreased the surface area of wounds that needed to be skin grafted |
| Jeschke 2006 / 1996-2006 | The purpose of the present study was to determine the effect of propranolol administration on infection, sepsis and inflammation in severely burned pediatrics patients | Patients younger than 18 years of age and TBSA burns of greater than 40% | Randomized controlled trial (SC) / Randomization list | 0.5-1.5mg/kg by mouth every 6 hours | Standard Care, not described by authors | Resting energy expenditure; infections episodes and sepsis; serum cytokines | No/ Yes/ Yes, described. No funding by pharmaceutical industry/ No | Non selective beta1/beta2 antagonist does not affect the incidence of infections or sepsis in severely burned pediatric patients, indicating that propranolol has no adverse effect on th eimmune system and function. We suggest that propranolol treatment is beneficial in burn victims and may improve survival |
| Herndon 2001 / January through December 1999 | Determine if beta-blockade with propranolol would decrease resting energy expenditure and muscle catabolism in patients with severe burns | Patients less than 18 years of age, with burns on more than 40% of TBSA and transferred to the hospital within one week after injury | Randomized controlled trial (SC) / Random number-generating scheme | Propranolol 1.98/kg/d | Standard Care, not described by authors | Heart rate, blood pressure, serum glucose, hormone values, potassium, occurrence of infections; energy expenditure, body composition | No/ Yes/ Yes, described. No funding by pharmaceutical industry/ No | Long-term beta-blockade decreases lean-mass catabolism in severely burned children. These changes would presumably improve the patients' strength and ability to recuperate. |

**Additional File 1: General Characteristics of included Studies.**
