# Supplementary material for: Safety and effectiveness of propranolol in severely burned patients: systematic review and meta-analysis
Source: World J Emerg Surg. 2017 Mar 2;12:11. doi: 10.1186/s13017-017-0124-7 (PMC5335497; doi:10.1186/s13017-017-0124-7)
Supplement: Additional file 2: — Appendix 1. Search strategy. (DOCX 51 kb) [file 13017_2017_124_MOESM2_ESM.docx]

**Appendix 1. Search Strategy**

Search strategy for Medline (Ovid):

|  |
| --- |

1.exp burns

2.burn$.mp

3.(burn$ adj2 injury).mp

4.(thermal adj2 injury).mp

5.or/

6. exp propranolol

7. propranolol.mp

8. (beta adj2 block$).mp

9. (beta adj2 antagonis$).mp

10.or/

11.exp randomized controlled trial

12.exp controlled clinical trial

13.randomized.ab.

14.trial.ab

15.or/

16. 5 and 10 and 15

**Embase:**

1. burns /exp

2.burn*:ti,ab

3.(burn* next/2 injury):ti,ab

4.(thermal next/2 injury):ti,ab

5.or/

6. propranolol /exp

7. propranolol:ti,ab

8. (beta next/2 block*):ti,ab

9. (beta next/2 antagonis*):ti,ab

10.or/

11. 'randomized controlled trial'/exp

12. 'controlled clinical trial'/exp

13. randomized:ti,ab

14. randomly:ti,ab

15. trial:ti,ab

16. or/

17. 5 and 10 and 16

Central (Through Ovid)

1.exp burns

2.burn$.mp

3.(burn$ adj2 injury).mp

4.(thermal adj2 injury).mp

5.or/

6. exp propranolol

7. propranolol.mp

8. (beta adj2 block$).mp

9. (beta adj2 antagonis$).mp

10.or/

11. 5 and 10
